# Application of Autoclave Treatment for Development of a Natural Wheat Bran Antioxidant Ingredient

**DOI:** 10.3390/foods9060781

**Published:** 2020-06-12

**Authors:** Daniel Rico, Adriana Villaverde, Cristina Martinez-Villaluenga, Angel L. Gutierrez, Pedro Antonio Caballero, Felicidad Ronda, Elena Peñas, Juana Frias, Ana Belen Martin Diana

**Affiliations:** 1 Agrarian Technological Institute of Castilla and Leon (ITACyL), Ctra. Burgos Km 119, Finca Zamadueñas, 47071 Valladolid, Spain; ricobdaniel@itacyl.es (D.R.); vilmonad@itacyl.es (A.V.); 2 Department of Food Characterization, Quality and Safety, Institute of Food Science, Technology and Nutrition (ICTAN-CSIC), Juan de la Cierva, 3, 28006 Madrid, Spain; c.m.villaluenga@csic.es (C.M.-V.); elenape@ictan.csic.es (E.P.); j.frias@csic.es (J.F.); 3 Department of Agriculture and Forestry Engineering, Food Technology, College of Agricultural and Forestry Engineering, University of Valladolid, Av. Madrid, 44, 34004 Palencia, Spain; anlugudelafu@gmail.com (A.L.G.); pedroantonio.caballero@uva.es (P.A.C.); mfronda@uva.es (F.R.)

**Keywords:** wheat bran, autoclave, antioxidant properties, phenolic compounds, techno-functional properties, ferulic acid

## Abstract

The study evaluated the effect of autoclaving as a hydrothermal treatment on the quality and bioactivity of wheat bran (WB) with the objective of producing a natural ingredient with enhanced healthy properties. Nutritional, antioxidant, techno-functional and sensorial parameters were studied, and temperatures of 100, 115 and 130 °C were explored. Of these, 130 °C was found to be the best treatment, resulting in an ingredient with high storage stability, antioxidant properties, a four-fold increase in the concentration of free ferulic acid (compared with non-treated WB), and increased content of apigenin-6-C-arabinoside-8-C-hexoside, a flavonoid with reported antioxidant and antifungal properties. On the other hand, the autoclave treatment enhanced water absorption capacity and reduced WB pasting viscosity, mainly at higher temperature (130 °C), which would allow incorporation of the treated WB in liquid matrices such as juices, soups or milkshakes, among others. Although the glycemic index (GI) of the autoclaved samples increased, the use of intermediate particle size of 106 to 300 µm could contribute to the reduction of the glycemic load.

## 1. Introduction

Consumption of nutraceutical ingredients is shifting towards naturally-occurring foods with high content in bioactive compounds, instead of fortified food or dietary complements. According to the Food and Agriculture Organization of the United Nations (FAO), micronutrient deficiencies, often called “hidden hunger,” affect one-third of world population [1].

Epidemiologic studies have reported that an intake of 14 g of dietary fiber per 1000 kcal would enhance heart health [2]. Furthermore, according to the Food and Nutrition Board of the Institute of Medicine [3], dietary fiber intake lowers the risk of coronary disease and cancer. Wheat bran (WB) is a natural ingredient, with excellent fiber content, and its consumption has increased gradually in recent years, probably due to the concern and awareness of consumers of the influence of food on health.

WB is a by-product of the wheat milling process. It is composed of more than 40% dietary fiber. It is also rich in vitamins, minerals and active compounds, such as alkyl resorcinols, ferulic acid, flavonoids, carotenoids, lignans and sterols [4,5,6]. Commonly, WB is produced by a roller milling process of the wheat kernels, resulting in easy separation of the bran and germ from the endosperm and, usually, bran fractions are removed with most of the bran layers intact. WB has been used for food [7] and non-food applications [4]; 90% is used as a livestock feed and only 10% is used in the food industry [8].

Phenolic acids (cinnamic and benzoic acids) are the most important phytochemicals found in the WB of cereal grains. Hydroxycinnamic acids include ferulic, sinapic and *p*-coumaric acids, among others [9]. Phenolic acids in WB occur in bound and free forms, and 95% of ferulic acid, the most abundant, is linked to cell wall polysaccharides [10]. The antioxidant value of WB is mainly evaluated in terms of its phenolic acid content [5,11,12,13,14].

Previous studies have reported that thermal processes such as autoclaving could be responsible for an increase in the content of free phenolic acids of the final product [15]. Hydrothermal treatment of cereals causes an increase in the phenolic compounds due to the increase in the solubilization of the cell wall components of endosperm and bran layers, and the release of phenolic compounds. Increased antioxidant capacity after thermal processing is due to the release of phenolics from the cell matrix, synergistic effects of phenolics and other phytochemicals, caramelization products, and chemical oxidation of phenolics [16].

In addition to it antioxidant capacity, WB can provide other positive effects on consumer health, such as the reduction of the glycemic index (GI) of foods. It is well known that foods with high GI are associated with chronic pathologies such as obesity, metabolic syndrome, and diabetes. In connection with the above, the food industry faces a difficult challenge to provide alternative products with low GI and high nutritional value. Therefore, the aim of the present study is to study the effect of autoclave treatments and particle size on phytochemical content, antioxidant activity, glycemic index, and physicochemical, techno-functional, morphological and sensorial evaluation of WB.

## 2. Materials and Methods

### 2.1. Raw Material

Wheat bran (WB) from winter wheat was kindly supplied by a local milling company (Emilio Esteban, Valladolid, Spain). The proportion of particle sizes between 1000 to 2000 μm was 99%.

### 2.2. Sample Preparation

Autoclaved samples. Unmilled WB as supplied was packaged in polyethylene bags and autoclaved at 100, 115 and 130 °C with pressure of 1400, 1600 and 2000 mbar and (15, 12 and 3 min, respectively) using air with an ILPRA Plus 100 equipment (Ilpra Systems, Barcelona, Spain).

After treatment, samples were coded (100 °C (AUT100), 115 °C (AUT115) and 130 °C (AUT130)) and stored at room temperature and under dark conditions to ensure stability of wheat bran until analysis. An untreated sample was used as control.

Milled samples. The autoclave treatment producing optimal effect on WB quality and antioxidant properties (130 °C) was applied to WB samples with different particle sizes. WB was milled with a Laboratory Mill 3100 (Perten Instruments, Hägersten, Sweden) to reduce the particle size until more than 95% of the bran particles of each sample passed through a 500-μm sieve. Bran was further sieved to obtain different particle size fractions, i.e., >300, 300–106 and <106 μm.

### 2.3. Physicochemical Properties of WB

#### 2.3.1. Proximal Composition

Moisture content was analyzed gravimetrically using drying treatment of the samples at 100 °C for 24 h [17]. Fat content was measured using petroleum ether 40–60 °C for over 4 h of extraction and gravimetrically determined. Nitrogen content was determined by the Dumas method (AOAC, 2005) and protein was calculated from nitrogen content using the conversion factor of 6.25. Ash content was determined following the method 923.03 (AOAC, 1990). Total dietary fiber was determined with an enzymatic method (985.29, AOAC, 1995). All the parameters were expressed as grams per 100 g of sample.

#### 2.3.2. Peroxidase (POD) Activity

POD activity was evaluated spectrophotometrically following the method of Sheu and Chen [18]. A double beam spectrophotometer (Shimadzu, 2101PC, London, United Kingdom) and a 1-cm path-length cuvette was used for enzymatic activity measurement. The spectrophotometer had a thermostat-controlled cell holder that maintained the temperature of the cuvette at 37 °C. POD activity was determined by measuring the increase in absorbance at 420 nm over time (one unit of enzyme activity is defined as an increment of 0.1 in absorbance per minute).

#### 2.3.3. Determination of Malondialdehyde (MDA)

Lipid oxidation was measured through the analysis of malondialdehyde (MDA) using the method described by Vyncke [19]. Results were expressed as mg of MDA per kg of WB sample.

### 2.4. Phenol Quantification and Characterisation

#### 2.4.1. Extracts

In order to increase the release of bioactive compounds, a sequential extraction method was used with acidified methanol and acetone, solvents that have shown appropriate extraction yields for cereal bran [20]. One gram of ground (<0.5 mm) sample was extracted with 10 mL of methanol:water (1:1, v:v; acidified to pH 2 with 0.1 M HCl) at controlled temperature (25 °C, 250 rpm and 1 h). After centrifugation (2800× *g*, 10 min), supernatant was separated, filtered (Whatman paper No. 1) and adjusted to 25 mL using the extracting solvent. A 10 mL volume of acetone:water (70:30, v:v) was added to the previous centrifugation pellet and the tube placed in a orbital-shaking temperature-controlled chamber (250 rpm, 25 °C, 20 min). After centrifugation (2800× *g*, 10 min), supernatant was collected, filtered (Whatman paper No. 1), and adjusted to 25 mL by extracting solvent added through the filter. This was then combined with the previous acidified extraction. Extract aliquots were stored at −80 °C.

In the case of the extracts used for quantification and identification of soluble phenolics, the extracts were obtained following the same described procedure but with no pH modification of the solvent (methanol:water).

#### 2.4.2. Total Phenols (TP)

TP were measured using Folin–Ciocalteu reagent, according to Slinkard and Singleton [21], with modifications [22]. The absorbance was measured at 765 nm with a microplate reader (Fluostar Omega, BMG, Ortenberg, Germany). Results were expressed as Gallic Acid Equivalents (GAE)/100 g dry sample.

#### 2.4.3. Determination of Ferulic Acid and Soluble Phenolic Compounds

Quantification of ferulic acid (FA) was determined in the extracts by high performance liquid chromatography (HPLC) with the Agilent 1200 HPLC series equipment (Agilent Technologies, Madrid, Spain) equipped with a quaternary pump, degasser, automatic injector and an array diode detector (DAD).

An aliquot (20 μL) was injected into a C18 reverse phase analytical column (150 × 4.6 mm internal diameter; particle size, 5 µm) (Teknokroma Analitica S.A., Barcelona, Spain) for separation at 25 °C. The binary mobile phase consisted of a 6% acetic acid in 2 mM sodium acetate buffer (Solvent A, pH 2.55, v:v) and acetonitrile (solvent B), and the isocratic gradient was Solvent A:Solvent B (85:15, v:v). The flow rate was 1.0 mL/min for a total run of 15 min. Ferulic acid standards were prepared in the range of 3.19 to 408.80 ppm for the calibration curve. All standards and samples were dissolved in methanol. The detection was carried out at 320 nm for ferulic acid. The R^2^ coefficient for the calibration curve was 0.999. Ferulic acid was identified by similar retention times and UV-vis spectra to those of standards. The ferulic acid content was expressed in mg/100 g dry sample.

Quantification and identification of soluble phenolic flavones, caffeic acid hexoside, and dihydroferulic acid were determined following the method described previously by García-Villalva et al. [23]. Identification of soluble phenols was carried out by high performance liquid chromatography (HPLC) using a 1100 series chromatograph coupled to a photodiode array detector (model G1315B, Agilent, Santa Clara, CA, USA) that were controlled by the Agilent software v. A.08.03 (Santa Clara, CA, USA). Samples (20 μL) were injected onto a C_18_ Kinetex-F5 column (5 μm, 250 × 4.6 mm; Phenomenex, Macclesfield, UK). Compounds were eluted from the column at a flow rate of 1 mL/min using 1% formic acid in water (mobile phase A) and acetonitrile (mobile phase B) and the following gradient program: from 5% B to 60% B in 37 min, from 60% B to 98% B in 3 min, from 98% B to 5% B in 5 min. The HPLC system was coupled to an ion-trap mass spectrometer using an electrospray interface (Agilent, Santa Clara, CA, USA). Analysis parameters were set up using a negative ion mode with spectra acquired over the range from 100 to 1200 m/z. The optimum values for the electrospray ionization mass spectrometer (ESI-MS) parameters were: nebulizer gas pressure of 65.0 psi, flow rate of nitrogen of 11 L/min, capillary temperature of 350 °C and voltage of 3.5 kV. Collision-induced fragmentation was carried out in the ion trap using helium and a collision energy of 50%. The phenolic compounds were identified according to their maximum absorbance at wavelengths 290 and 320 nm, molecular mass and fragmentation pattern. Quantification was made by external calibration curves in the concentration range between 1 and 100 µg/mL of standard compounds: dihydroferulic acid, caffeic acid and apigenin 8-*C*-glucoside (Sigma-Aldrich, Madrid, Spain). Analyses were performed in duplicate and results were reported as μg/g of WB on a dry weight basis (dw).

### 2.5. Total Antioxidant Capacity (TAC)

TAC was measured on the methanolic extracts using 2,2-Diphenyl-1-picrylhydrazyl (DPPH) radical scavenging activity, Oxygen Radical Absorbance Capacity (ORAC), Ferric Reducing Ability of Plasma (FRAP) and 2,2′-Azinobis-(3-ethylbenzothiazoline-6-sulfonate (ABTS) methods. In addition, DPPH and ABTS methods on solid samples without previous extraction (Q-DPPH and Q-ABTS) were carried out. Reagents were provided by Sigma-Aldrich (Madrid, Spain).

#### 2.5.1. DPPH and Q-DPPH Assay

The antioxidant activity of the extracts against the DPPH radical was estimated according to the procedure described by Brand-Williams et al. [24] with modifications. An amount of 25 µL of extracts was mixed with 100 µL of MilliQ water and 125 µL of DPPH working solution (100 µM using methanol as solvent) in a 96-well microplate. Absorbance at 515 nm was recorded for 30 min in a microplate reader (Fluostar Omega, BMG Ortenberg, Germany). Results were corrected for moisture and expressed as mg Trolox equivalent/100 g sample.

The Q-DPPH method was evaluated following the procedure describe by Serpen et al. [25], with modifications. Ten milligrams of powdered samples (particle size below 300 µm) were mixed with 1.5 mL of 60 µM DPPH methanolic solution. After incubation at 700 rpm for 30 min (Thermomixer Compact, Eppendorf AG, Hamburg, Germany), samples were centrifuged at 14,000× *g* for 2 min and the absorbance measured at 515 nm in a microplate reader (Fluostar Omega, BMG Ortenberg, Germany). Results were corrected for moisture and expressed as µmol Eq. Trolox/100 g of WB on a dry weight basis (dw).

#### 2.5.2. ORAC Assay

The procedure was based on a previously reported method with slight modifications [26]. The standard curve of Trolox (15–240 mM) and samples were diluted in phosphate buffer (10 mM, pH 7.4). A volume of 150 μL fluorescein was placed in 96-well black polystyrene plates, and 25 μL of Trolox standard, sample or phosphate buffer as blank were added, all in duplicates. Samples, standards and blanks were incubated with fluorescein at 37 °C for 3 min before 2,2′-azobis (2-methyl propionamidine) dihydrochloride solution was added to initiate the oxidation reaction. Fluorescence was monitored over 35 min with a microplate reader (Fluostar Omega, BMG Ortenberg, Germany), using 485 nm excitation and 528 nm emission filters. Results were calculated using the areas under the fluorescein decay curves, between the blank and the sample, and expressed as µmol Eq. Trolox/100 g of WB on a dry weight basis (dw).

#### 2.5.3. ABTS and Q-ABTS Assay

Antioxidant capacity against diammonium salt of ABTS radical was evaluated following the method first described by Miller and Rice-Evans [27], as modified by Martin-Diana et al. [22]. The absorbance was measured at 730 nm with a microplate reader (Fluostar Omega, BMG Ortenberg, Germany). Results were corrected for moisture and expressed as Trolox equivalent (µg TE/g). The Q-ABTS method described by Serpen et al. [25], as modified in Martin-Diana et al. [22], was used to evaluate the direct antioxidant capacity of wheat bran samples. Results were corrected for moisture and expressed as µmol Eq. Trolox/100 g of WB on a dry weight basis (dw).

#### 2.5.4. FRAP Assay

The assay was performed following the protocol reported by Vijayalakshmi and Ruckmani [28]. Results were expressed as nmol Fe^2+^/g of WB on a dry weight basis (dw).

### 2.6. Glycemic Index (GI) of WB

The GI in WB was determined by, first, measuring the content of available starch using the total starch assay kit of Megazyme (K-TSTA 08/16). Then, the in vitro starch hydrolysis rate was determined as described by Gularte and Rosell [29]. Glucose analysis was performed using a GOPOD kit (Megazyme, Bray, Ireland). Hydrolysis index (HI) and glycemic index (GI) values were calculated as proposed by Granfeldt [30].

### 2.7. Techno-Functional Properties of WB

#### 2.7.1. Hydration, Foaming and Emulsifying Properties

Water absorption capacity (WAC, g H_2_O absorbed/g sample), water absorption index (WAI, g gel/g sample), water solubility index (WSI, g soluble matter/100 g sample) and swelling power (SP g gel/g insoluble matter) determinations were made following the procedure described by Villanueva et al. [31]. Oil absorption capacity (OAC, g oil absorbed/g sample), emulsion activity (EA, mL emulsified layer/100 mL of the emulsion before centrifugation) and emulsion stability (ES, mL emulsified layer after heating/100 mL of the emulsion before centrifugation) were measured by the method described by Kaushal et al. [32]. Foaming capacity (FC, mL foam/g sample) and foam stability (FS, mL foam after storage/100 mL of foam freshly prepared) were established as described by Collar and Angioloni [33]. Results were referred to dry matter to avoid the effect of different water content in the samples.

#### 2.7.2. Least Gelation Concentration (LGC)

The least gelation concentration (LGC) was calculated according to the method of Acevedo et al. [34] with slight modifications. Tubes containing 2 mL of milled bran suspensions in distilled water at different concentrations (2–25%, w:v) were heated for 1 h in a boiling water bath. Then, they were rapidly cooled under tap water and kept for 2 h at 3 °C. The LGC was determined as the concentration at which the sample did not slip after inverting the test tube.

#### 2.7.3. Pasting Properties

The pasting properties were determined from viscometric tests using a Kinexus Pro + rheometer (Malvern Instruments Ltd., Malvern, UK) with a starch pasting cell geometry and following AACC International Method 76-21.01 Standard No. 2 [35], with slight modifications: the concentration of WB was increased up to 20%. The R Space ver. 1.72 software (Malvern Instruments Ltd., UK) was used to obtain the pasting temperature (PT), pasting time (Pt), peak viscosity (PV), trough viscosity (TV), breakdown (BD), final viscosity (FV) and setback (ST).

#### 2.7.4. Rheological Properties: Viscoelastic Behavior of Gels

Gels obtained from viscometric tests were transferred and placed between parallel plate geometry (40 mm diameter) of Kinexus Pro + rheometer (Malvern Instruments Ltd., UK) to perform the dynamic oscillatory test. Samples were left to rest for 5 min to allow relaxation. The temperature was set at 25 °C with a Peltier plate controller. Frequency sweeps were performed from 10 to 1 Hz in the linear viscoelastic region (LVR), which was previously determined in a strain sweep test was carried out from 0.01 to 100 % at 1 Hz. A fixed shear strain value was selected to perform the frequency sweeps (0.1 %) for all the samples. The maximum stress (τmax) within the LVR was also determined as the decrease of the elastic modulus (G′) above 10%. Frequency sweep data were fitted to potential equations described by Abebe and Ronda [36]. Gel samples were made in duplicate and rheological analysis of the gels was also carried out in duplicate.

#### 2.7.5. Scanning Electron Microscopy (SEM)

Scanning electron microscopy was evaluated using a scanning electronic microscope (FEI QUANTA 200, Graz, Austria). Images were taken using 150× and 1000× magnifications for surface and section. Voltages between 5 and 15 kV were used depending on the detector and the topography of the sample and the spot sizes.

### 2.8. Organoleptic Properties of WB

#### 2.8.1. Colorimetric Analysis

Lightness (L*), redness (a*) and yellowness (b*) were measured using a colorimeter (Konica Minolta, CM-2600d, Osaka, Japan). The illuminant was D65 (color temperature of 6504 K). The colorimeter was standardized using a light trap and a white calibration plate. Measurements were taken on the samples packaged at 10 different points.

#### 2.8.2. Sensory Analysis

A panel of eight panelists with previous experience in sensory analysis, aged between 20 and 45 years old, were recruited from ITACyL Staff. Training was carried out in common sessions, using a range of commercial bran products in order to establish consensus on the descriptors. Color intensity, texture (looseness), aroma and acceptability descriptors were evaluated using a 9-point scale, with 1 = minimum and 9 = maximum intensity. Each judge was presented with a complete set of samples with random 3-digit codes for evaluation, generating one vector of multiple dependent data as shown by Carabante and Prinyawiwatkul [37].

### 2.9. Statistical Analysis

The effect of autoclave treatments and particle size on the parameters studied was evaluated and one-way ANOVA was applied. All statistical analyses were performed using Statgraphics Centurion XVI^®^.

## 3. Results and Discussion

### 3.1. Thermal Treatment and Particle Size on Physicochemical Properties of WB

Proximal composition of WB generated in the milling process of wheat grains showed that this ingredient has important nutritional value since it is abundant in dietary fiber and protein as main components, with fat and ash in minor proportions (Table 1). The fat content of WB was 4.32 g/100 g, in agreement with those reported by Curti et al. [7] (3.9 to 8.1 g/100 g). The fatty acid profile corresponded mainly to saturated (from 18 to 37 g/100 g), followed by monounsaturated fatty acids (13 and 28 g/100 g) [38].

The whole wheat bran in the present study had a high dietary fiber content, 36.8 g/100 g, similar to the values reported by D’hoe et al. [39] (38 g/100 g) and Curti et al. [7] (33.4 g/100 g), and lower than those reported by Chalamacharla et al. [40] and Onipe et al. [41], who found fiber content higher than 50 g/100 g. WB fiber is composed basically of arabinoxylan, cellulose, lignin, fructans and mixed linked β-glucans [4,41].

The ash content of WB was 4.93 g/100 g, similar to values described by other authors (5.1 g/100 g) [39]. The particle size significantly affected the proximal composition. Size reduction of WB from coarse particles to fine particles significantly decreased dietary fiber (44.8 and 26.1 g/100 g, respectively) and increased the protein content (17.38 and 18.56 g/100 g, respectively), as expected, without significant differences in other proximal parameters analyzed.

**Figure 1:**
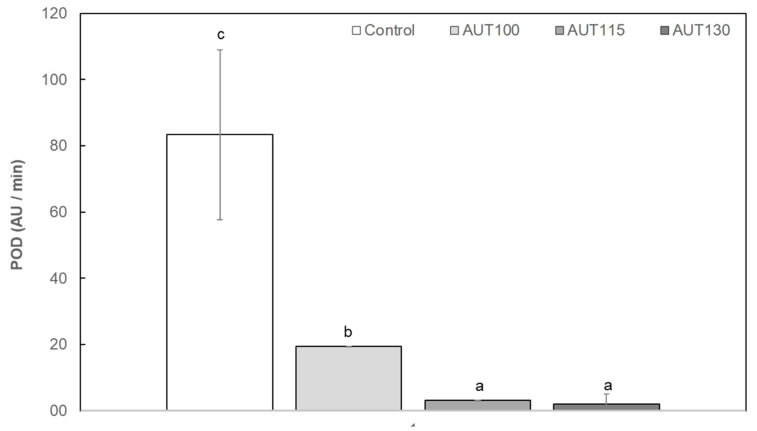
Peroxidase activity (POD) of wheat bran (WB) Control, AUT100, AUT115 and AUT130. POD values are expressed as AU/min. Values with different small letters show significant differences (*p* < 0.05) between autoclave treatments.

The effect of thermal treatment on peroxidase (POD) activity and malondialdehyde (MDA) content was evaluated as parameters related with the quality and stability of the ingredient. POD, a heat-stable enzyme, was evaluated as an indicator of the efficacy of thermal treatment for enzymatic inactivation. POD inactivation reduces the susceptibility of WB to rancidity and enhances its stability during its shelf life [42]. The results showed a significant effect of autoclaving on the POD activity of WB (Figure 1).

POD activity was significantly reduced with the application of heat treatments of 100 °C by approximately 75% which is clearly insufficient to guarantee lipase inactivation. On the other hand, inactivation levels of 90 and 98% were observed when the treatment temperature was increased to 115 °C and 130 °C, respectively.

**Figure 2:**
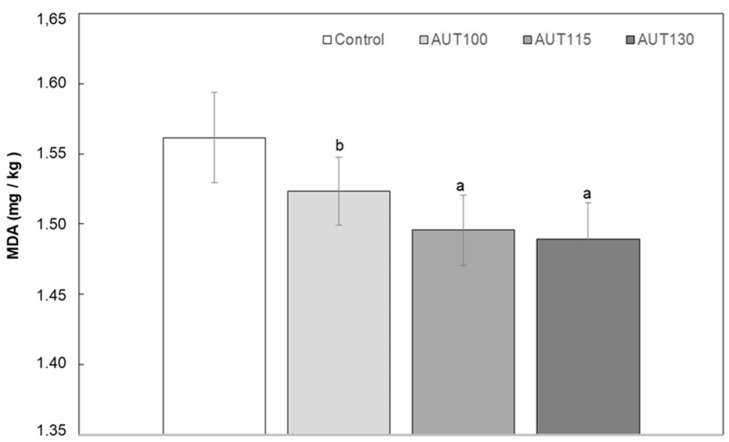
Malondialdehyde (MDA) of WB Control, AUT100, AUT115 and AUT130. MDA values are expressed as mg/kg of WB. Values with different small letters show significant differences (*p* < 0.05) between autoclave treatments.

Lipid oxidation is one of the major causes of losses in food quality, especially in those containing polyunsaturated fatty acids. Unsaturated fatty acids are oxidized to form odorless, tasteless hydroperoxides. The formed hydroperoxides are further decomposed to secondary oxidation products, which are mainly aldehydes, such as hexanal, 4-hydroxynonenal, and MDA. The present study evaluated the effect of thermal treatment on MDA levels in WB (Figure 2) as an indicator of quality. The results showed that the content of MDA was reduced significantly (*p* < 0.05) with autoclave treatment. Control and WB treated at 100 °C showed significantly higher values than WB treated at 115 and 130 °C, with these two samples showing similar MDA levels. This decrease in MDA can likely be associated with the inactivation of lipases that occurs during the hydrothermal treatment [43].

### 3.2. Effect of Thermal Treatment on Phenolic Profile of WB

**Figure 3:**
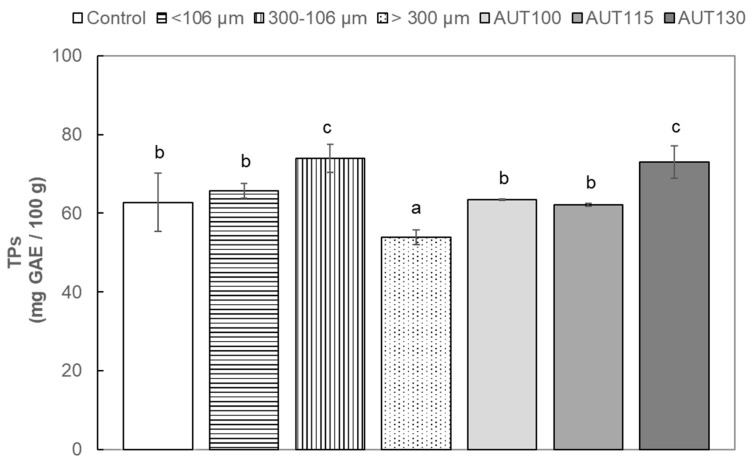
Total phenols (TPs) of WB Control, AUT100, AUT115, AUT130 and WB autoclaved at 130 °C with different particle size, >300, 300–106, and <106 µm. TP values are expressed as mg GAE/100 g. Values with different small letters show significant differences (*p* < 0.05) between treatments.

Figure 3 shows the total phenolic content (TP) of WB extracts measured using the Folin–Ciocalteu method. Total phenolic content in methanolic extract from untreated WB (control) (62.8 mg GAE/100 g) were within the range reported by other authors (40 to 74 mg GAE/100 g) [38,44]. A significant (*p* < 0.05) increase to nearly 10% in TP was exclusively observed at the highest temperature (130 °C). This effect can be associated with the release of phenolic compounds covalently bound to cell wall polysaccharides or accumulated in the cellular vacuoles [45,46]. The particle size of WB showed a significant effect on total phenolic content of WB. Intermediate particle size between 300 and 106 µm showed the highest TP content (73.9 mg GAE/100 g), followed by WB fraction with finer particle size (<106 µm); these results agree with other authors [13,47,48]. This observation may be partially due to a more efficient extraction of free phenolic compounds from WB particles with a higher surface area/volume ratio.

The phenolic profile of WB and the effect of autoclave treatment on its phenolic profile are shown in Table 2 and Supplementary Table S1. The phenolic fraction of untreated WB (control) was composed of hydroxybenzoic acid (caffeic acid *O*-hexoside, ferulic, and dihydroferulic) and flavone (apigenin *C*-glycosides) phenolic classes in agreement with previous studies [49,50]. Ferulic acid was the most abundant phenolic compound present in untreated WB (52% of the total compounds). Apigenin-6,8-*C*-pentoside-8,6-*C*-hexoside isomer 1 and 2 were the second most important compounds (14% and 18% of total phenolic compounds, respectively) followed by minor amounts of caffeic acid *O*-hexoside, apigenin-6-*C*-arabinoside-8-*C*-hexoside and dihydroferulic acid (6%, 4% and 4% of the total phenolic compounds, respectively).

**Scheme 1:**
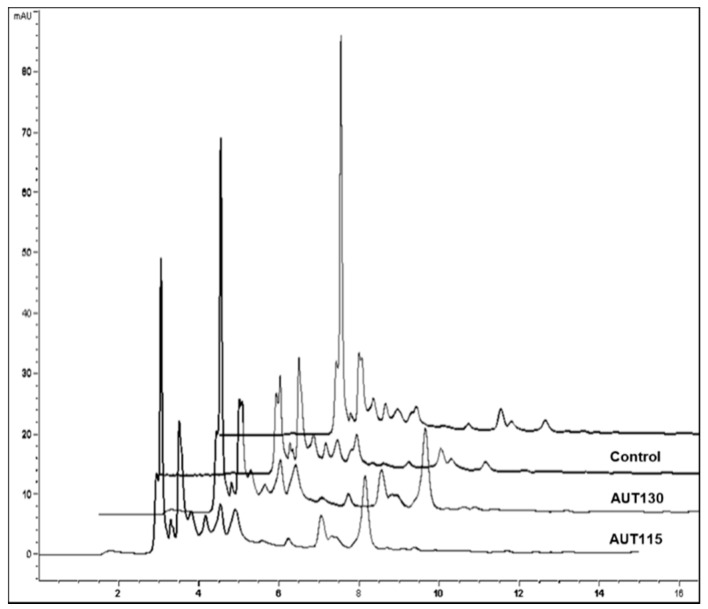
Ferulic acid (FA) profile of Control, AUT100, AUT115, and AUT130.

Differential effects of thermal treatment were observed among phenolic compounds (Table 2). Free ferulic acid increased exclusively when WB was heated with temperatures above 100 °C (Scheme 1). The highest ferulic acid increase was found for AUT130 (four-fold higher content vs. control). Similarly, autoclaved WB showed 1.4 and two-fold higher content of caffeic acid *O*-hexoside and dehydroferulic acid, without significant differences among thermal treatments (*p* ≥ 0.05). Consistent with our results, Calinoiu and Vodnar [50] reported a general increase of phenolic acids (di-OH-benzoic, caffeic, vanillic, *p*-coumaric, sinapic and ferulic acids) in WB treated at 80 °C for 10 min. A high proportion of phenolic acids present in WB are covalently bound to arabinoxylans, cellulose and lignans [9]. Thermal hydrolysis of ester linkages between these phenolic acids and polysaccharides was reported by Liao et al. [51], which could explain the increased amounts of phenolic acids in WB after autoclave processing applied in this work. Moreover, hydrothermal processing may cause a disruption of the cell membranes and vacuoles that make phenolic compounds more available the cell wall components.

Flavonoid glycosides varied in their thermal stability (Table 2). In accordance with previous studies focused on apigenin-6-*C*-arabinoside-8-*C*-hexoside, it increased after thermal treatments; the highest increase was observed for AUT100 and AUT130 (1.8 and 1.9-fold higher content vs. control, respectively). The opposite effect was found for apigenin-6,8-*C*-pentoside-8,6-*C*-hexoside isomers, which were significantly reduced after autoclaving. Regarding total content of flavones, thermal treatment had an overall negative effect on apigenin *C*-glycosides. Unlike our results, Calinoiu and Vodnar [50] reported higher amounts of apigenin glucosides after thermally treating WB at 80 °C for 10 min. Differences in the effect of thermal treatment on WB apigenin glucosides between our results and previous studies could be due to differences in the thermal conditions applied. The higher temperatures used in the present study ranging from 100 to 130 °C could have induced thermal degradation of apigenin-6,8-*C*-pentoside-8,6-*C*-hexoside isomers. It is worth mentioning that losses in the content of apigenin *C*-glycosides in the present study were less pronounced for AUT130 (68% and 39% reduction vs. control for apigenin-6,8-*C*-pentoside-8,6-*C*-hexoside i1 and i2, respectively) compared to AUT115 and AUT100. These results could be related to differences in holding time among autoclave treatments as observed by Hostetler et al. [52], who investigated the stability of apigenins to autoclave processing in celery and chamomile. In WB AUT130, a short holding time (3 min) could lead to lower degradation of apigenin *C*-glycosides compared with AUT115 and AUT100, in which thermal treatment was set at 115 °C for 12 min and 100 °C for 15 min, respectively.

### 3.3. Effect of Thermal Treatment and Particle Size on Total Antioxidant Capacity (TAC) of WB

TAC analyses evaluating radical scavenging and reducing capacities were carried out with the objective to quantify the effect of autoclaving and particle size on final antioxidant properties of WB. In order to obtain a global assessment of antioxidant capacity in WB, a matrix with a high fiber content, and ensure the evaluation of the synergistic action of all dietary antioxidants, extractable and direct methods were measured.

**Figure 4:**
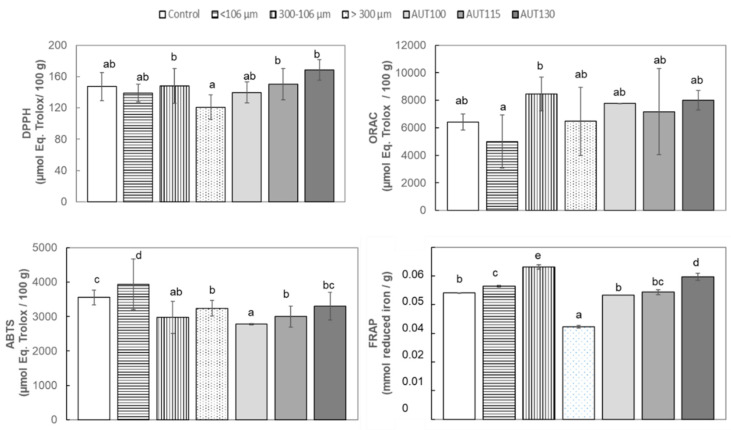
DPPH, ORAC, ABTS and FRAP of WB Control, AUT100, AUT115, AUT130 and WB autoclaved at 130 °C with different particle size, >300, 300–106, and <106 µm. Values are expressed as: µmol Eq. Trolox/100 g for ORAC, DPPH, ABTS, and mmol Fe^2+^/g for FRAP. Values with different small letters show significant differences (*p* < 0.05) between treatments.

The ability to scavenge the DPPH radical was analyzed in control WB and WB treated at different temperatures. The results indicated than WB treated at 130 °C showed higher ability than WB treated at 115 °C and both were significantly higher than in WB non-treated or treated at the low temperature of 100 °C (Figure 4). The same behavior was observed when Q-DPPH was evaluated, with the highest values appearing in samples treated at 130 °C followed by samples at 115 °C. The higher antioxidant ability observed in Q-DPPH vs. DPPH is probably based on the higher proportion of bounded fiber phenols non-extractables and measurables in the classical methods. The particle size also showed a significant (*p* < 0.05) effect on DPPH activity; in both methods the fraction with higher particle size showed higher antioxidant capacity than fractions with smaller particle size. Similar results were reported by Brewer et al. [13], who observed that the fine fraction showed the lowest DPPH activity.

**Figure 5:**
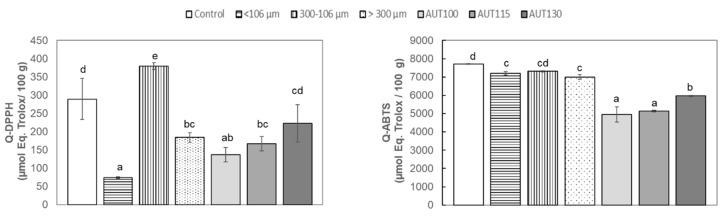
Q-DPPH and Q-ABTS of WB Control, AUT100, AUT115, AUT130 and WB autoclaved at 130 °C with different particle size, >300, 300–106, and <106 µm. Values are expressed as µmol Eq. Trolox/100 g. Values with different small letters show significant differences between treatments (*p* < 0.05).

Thermal treatments also produced an increment in ORAC values, and the highest were observed at 130 °C (Figure 4). Particle size also produced a significant effect on ORAC; the intermediate fraction (300–106 µm) contained higher antioxidant activity than compounds present in the fine fraction (Figure 5).

The ability to reduce Fe^3+^ to Fe^2+^ was evaluated on WB extract for different autoclave temperatures and different WB particle size using FRAP assay. FRAP values showed that fine particles demonstrated higher ability to reduce iron than coarse particles (Figure 5), opposite to the results observed by Brewer et al. [13]. Temperature again caused a significant enhancement of the activity, with autoclaving at 130 °C the most effective treatment for WB.

ABTS direct and indirect assays showed lower values in autoclaved samples, in contrast with the others markers analyzed. (Figure 4). Although ABTS values were higher in non-autoclaved samples, a significant effect of temperature was observed with increasing antioxidant capacity (ABTS) due to increasing treatment temperature. Regarding a particle size effect on ABTS antiradical activity, WB with finer particle size (<106 µm) showed the highest ABTS values for indirect method, and no significant differences due to particle size were observed in the case of indirect Q-ABTS method results (Figure 5).

### 3.4. Effect of Thermal Treatment and Particle Size on Glycemic Index (GI) of WB

**Figure 6:**
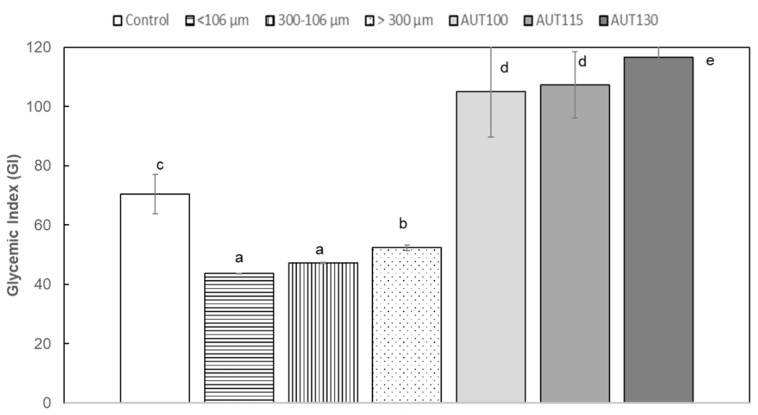
Glycemic index (GI) of WB Control, AUT100, AUT115, AUT130 and wheat bran autoclaved at 130 °C with different article size, >300, 300–106, and <106 µm. Values with different small letters show significant differences between treatments (*p* < 0.05).

The GI has proven to be a more useful nutritional marker than the chemical classification of carbohydrate (as simple or complex, as sugars or starches, or as available or unavailable), providing new insights into the relationship between foods and health. The GI indicates the rate at which 50 g of carbohydrate in a particular food is absorbed into the blood stream as blood sugar and ranges from 1 to 100. Foods can be classified by their GI, according to Gordillo-Bastidas et al. [53] as follows: (i) high GI foods (>70), (ii) medium GI foods (56–69) and (iii) low GI foods (<55). In the present study, the temperature produced a significant increase in GI index (Figure 6), probably due to the increment in hydrolysis of the carbohydrates, which increases the bioavailability of carbohydrate to hydrolysis. WB treated at 130 °C showed the highest GI, which might be associated with the gelatinization of the starch due to heat treatment, which has a linear and direct effect on GI (Figure 6). Moreover, the reduction in particle size decreased the sample GI; this effect is probably associated with a lower concentration of B-type starch in the fine fraction compared to the coarse fraction (Figure 6).

### 3.5. Effect of Thermal Treatment and Particle Size on Techno-Functional Properties of WB

#### 3.5.1. Hydration, Foaming and Emulsifying Properties

Results of functional properties of thermal treated samples are reported in Table 3. The samples autoclaved at 115 and 130 °C showed a significant (*p* < 0.05) increase in water absorption capacity (WAC) compared to the untreated sample, probably associated with an increase in the capacity of bran to bind water strongly through hydrogen bonds or nanopores present in the bran matrix or, in a weaker way, through micropores and stacking phenomena [54]. Some studies revealed no significant changes in water retention capacity when bran samples were processed under both dry and wet heat treatments such as extrusion, extrusion-cooking, autoclaving or steam cooking [54,55,56]. However, other authors have also reported an increase in water binding capacity in bran samples upon heat treatments such as boiling [56]. Wang et al. [54] described that an increase in water absorption capacity could be ascribed to residual gelatinized starch from endosperm present in bran samples and the ability of the starch to contribute to the bran hydration capacity. Thus, the different results found could be attributed to different starch content in the samples. The oil absorption capacity (OAC) of the samples was not affected by autoclave treatments. Contradictory results were observed by Arrigoni et al. [55], who detected an increase in the oil adsorption capacity of wet heat-treated wheat bran samples but not when samples were treated by dry heat. These results were attributed to structural surface changes, which in part result from starch gelatinization. Autoclave treatments did not significantly affect the water absorption index (WAI) and swelling power (SP) of bran samples. However, the water solubility index (WSI) significantly (*p* < 0.05) decreased in the samples autoclaved at 100 °C and 115 °C.

All treated samples exhibited a significant (*p* < 0.05) decrease in foaming capacity (FC) and the total loss of emulsion activity (EA). Drops in FC of 67% for bran autoclaved at 100 °C and of more than 90% for bran treated at 115 °C and 130 °C were observed. Similar behavior was previously observed for wheat gluten [57] and soy flour [58] foaming capacity, as a result of thermal treatment. The denaturation of proteins as a result of the heat treatment seems to explain these functional changes [57,58,59,60].

#### 3.5.2. Least Gelation Concentration (LGC)

The LGC values (Table 3) of all bran samples, including the control bran, were between 15 and 17 g/100 mL. Kausahl et al. [32] explained that the gelation capacity of flours is affected by the water competition between protein gelation and starch gelatinization. Although the ground materials in this paper were rather different than the samples used in the study of Kaushal et al. [32], as the starch content in the bran particle was probably low, LGC values would be more dependent on the protein content of the bran samples. In this way, autoclave treatments applied did not seem to affect the protein gelation properties.

#### 3.5.3. Pasting Properties

Pasting properties and viscosity profiles are shown in Table 4 and Supplementary Figure S1, respectively. Autoclave treatment modified the pasting profiles of bran. Pasting temperature increased in treated WB samples (73–74 °C) with respect to the control (70 °C). No significant (*p* < 0.05) differences were found among the treated samples. Similar behavior was found in the study of Bucsella et al. [56] analyzing the viscometric behavior of a hydrothermal-treated aleurone-rich wheat fraction. They attributed this increase to the heat treatment, which might modify the water binding ability of the bran constituents delaying the swelling of the starch. The presence of pre-gelatinized starch in the hydrothermal treated samples and the natural presence of dietary fiber as arabynoxylans, galactomannans and B-glucans, can impair the gelling ability of the starch by lowering the water availability for gel formation. The peak viscosity (PV) decreased with the temperature of treatment. The lowest value, 30% below that of the control sample, was obtained by the sample autoclaved at 130 °C. This decrease in PV was previously reported for similar heat-treated systems [56]. Bucsella et al. [56] explained this effect by the physical and chemical alteration of protein and carbohydrate composites and the formation of an insoluble matrix with reduced swelling capacity during the heat treatment.

Treated samples showed lower breakdown viscosity (BV) values than the untreated sample. This means a higher stability of the paste versus heating and stirring. This stability could be promoted by the presence of rigid, swollen starch granules and fiber composites that would remain unchanged after heating period applied during the test [56].

Samples treated at 115 and 130 °C showed significantly (*p* < 0.05) higher setback viscosity (SV) values. The same result was observed by Abdel-Haleem [61] in blends of dry-heat-treated wheat bran with refined wheat flour, where the modification of protein, starch and pentosan structure during the heat treatment would explain the formation of a highly rigid gel in the cooling phase and the increase in the setback viscosity of treated samples.

#### 3.5.4. Rheological Properties: Viscoelastic Behavior of Gels

The viscoelastic properties of gels made from the bran samples are shown in Table 4. The mechanical spectra of these gels are shown in Supplementary Figure S2. Dynamic moduli of studied systems fitted well to the power law model, as reflected in the high correlation coefficients obtained, which were also between 0.998 and 0.999. All analyzed samples exhibited higher elastic (G′) than viscous (G′′) modulus, with loss tangent values at 1 Hz well below 1 (0.206–0.224); thus, gels exhibited a solid-like behavior. Autoclave treatment significantly affected the viscoelastic properties of bran gels. The heat treatment led to a slight decrease of the elastic behavior of gels, which showed higher loss tangent values (6–9%) than the control gel. The maximum stress that gels were able to hold until the structure began to collapse (τ_max_) also decreased significantly in autoclaved bran gels (32%), revealing a weaker structure than that of the gels made with untreated wheat bran (see Table 4). The same trend was observed in the stress of the cross-point obtained from strain sweeps (G′ = G). No significant differences were observed in these rheological parameters among the treated samples. The viscoelastic moduli of gels were hardly affected by the treatment. Only the gel made from the AUT100 sample showed elastic and viscous moduli smaller than those of the control gel (37% and 33%, respectively) denoting a significantly lower consistency than the other gels. The variability in gel consistency may be attributed to fibril entanglement [62] and formation of protein aggregates or protein–fiber complexes [56] due to autoclave treatment. The heating rate may also affect the aggregation kinetics resulting in gels with different network structure (different pore size, strand thickness, or curvature) affecting viscoelastic moduli [63]. Gels with curved strands have lower G′ values than gels with predominantly straight strands, because bending deformation involves less energy than stretching deformation, and thin strands are easier to bend than thick strands (lower G′). The exponent “a”, which quantifies the dependence of G′ on frequency, did not vary among the different gels (0.15) and was always below the exponent “b” (0.28–0.32). Consequently, a positive “c” value (0.13–0.17) was always obtained, confirming all gels increased their viscous behavior with frequency. The exponents “b” and “c” increased significantly in the AUT100 gel, indicating that its G’’ and tan (δ)_1_ values increased faster with frequency than in the remaining gels.

#### 3.5.5. Scanning Electron Microscopy (SEM)

**Scheme 2:**
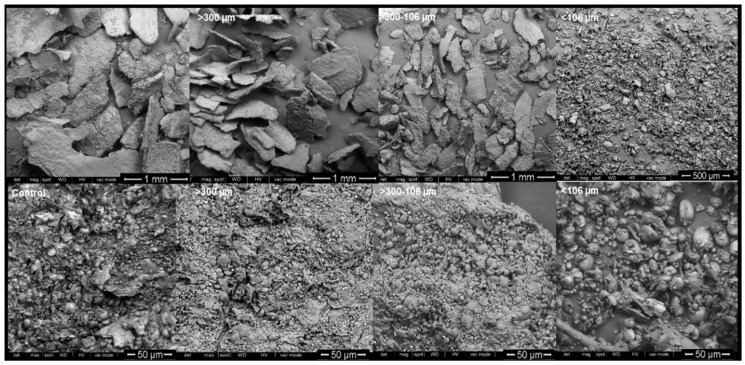
SEM images of WB Control, AUT100, AUT115, AUT130 and WB autoclaved at 130 °C with different particle size, >300, 300–106, and <106 µm.

The effect of thermal treatment and particle size on morphological properties of WB was analyzed using SEM. The starch granules observed showed typical type A and type B structures and, in samples with particle size above 106 µm, starch granules appeared closely associated with cellular structures (Scheme 2). From microstructure images, it appears that samples smaller than 106 µm may have suffered starch looseness and disassociation from fiber structures to a higher degree than in samples with higher particle size. In addition, fewer micropores seemed to appear in samples with particle size below 106 µm; these micropores correspond to hollow pericarp cells and spacing between histological layers [64], and these results support the importance of considering bran particle size fractionation in future applications, as this may affect physical properties of WB [62,63,64,65].

**Scheme 3:**
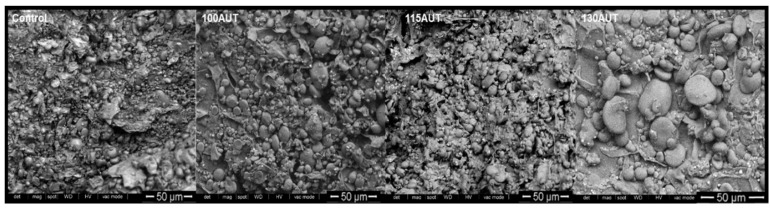
SEM images of WB Control, AUT100, AUT115 and AUT130.

The microstructure of samples treated with autoclaving can be observed in Scheme 3. Starch gelatinization may be responsible for the differences observed between untreated and autoclaved WB, regarding the aspect and texture of the starch granules, where in untreated samples show a much coarser aspect. Starch morphology appears to change from a more flat-like shape of type A starch granules (bigger granules) in samples untreated, to a round-like shape in 100 °C and 115 °C-treated samples. In those samples subjected to 130 °C autoclave treatment, the changes of the starch granule shape seem to have been limited, which may be due to the much shorter treatment applied in this case.

Moreover, in control WB the structure of the fibers is more appreciable in contrast with autoclaved WB, where agglomerated smooth surface structures can be observed, and, in all cases, the spherical and ovoid granules of wheat starch can be clearly observed associated with fiber.

Proteins, spherical in shape, can also be observed with the starch granules. However, the autoclaved bran internal structure is considerably different from that of the untreated WB, which probably corresponds to the fusion between starches, fiber and proteins, indicating the pre-gelatinization of the starch containing the bran.

### 3.6. Effect of Thermal Treatment and Particle Size on Colorimetric Properties of WB

WB was also evaluated using color coordinates (CIE LAB*). Results showed a significant effect on WB color depending on its particle size, from coarse particles to fine particles, during milling and sieving (Table 5).

Luminosity values ranged from 64.48 in raw WB to 83.55 in WB fine particles. The b* coordinate also showed the same trend with lowest values in WB at fine particle size (13.03 vs. 17.94). The increase of L* and the reduction of b* might be associated with its floury nature as a result of the residual endosperm from the inner pericarp found in the WB during the sieving. As expected, hue increased significantly while chroma decreased with reduction of particle size in WB.

The autoclave treatment also produced a significant effect on WB color. The decrease in luminosity was lowest for WB treated at high temperature. In contrast, those samples showed the highest b* values due to the possible association with colored products from the Maillard reaction.

### 3.7. Effect of Thermal Treatment on Sensorial Properties of WB

**Figure 7:**
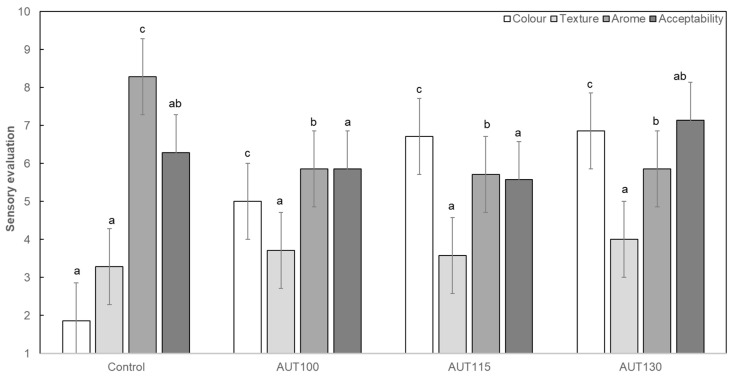
Sensory evaluation of WB Control, AUT100, AUT115 and AUT130. Values with different small letters show significant differences between autoclave treatments (*p* < 0.05).

The sensory scores of the WB are shown in Figure 7. There was a significant (*p* < 0.05) difference between the WB treated with autoclave in terms of color, aroma, and overall acceptability, with the latter determined on the basis of quality scores obtained from the evaluation.

The panelists detected a significant (*p* < 0.05) change in color between the untreated and autoclaved WB samples, and this modification was more evident when higher temperatures were used in the process. This modification was associated with the Maillard reaction produced during the autoclave treatment.

However, the panelists could not appreciate differences in texture, although the autoclave process modified the internal structure of WB, according to SEM (Scheme 3). The aroma was also modified by the treatment, with the autoclave reducing the typical aroma associated with the wheat bran. Regarding overall acceptability, the panelists scored all WB samples with similar values, with the sample treated at 130 °C receiving scores closer to that of the control and higher than those of the samples treated at 100 and 115 °C; this was probably associated with the shorter times although temperatures were higher.

## 4. Conclusions

This study showed that the use of autoclaving enhanced the antioxidant properties of WB and the bioavailability of certain types of non-free phenol compounds, such as ferulic acid, and other compounds of interest, such as flavonoids (glycosylated apigenin). WB particle size had an effect on bioactive properties and, with the aim of finding a balance between quality and bioactive aspects, WB fractionated with intermediate particle size, in the range of 300–106 µm, and treated at 130 °C, appears to be the most appropriate. Since the hydrothermal process did not produce noticeable sensory changes in WB, these results indicate that autoclaved WB may be used as an ingredient to be incorporated in drinkable products, such as juices or milkshakes, without modifying sensory and rheological properties following the common thermal processes that these products require for stabilization.

## Figures and Tables

**Table 1:** Proximal composition of control wheat bran (WB); values are expressed as g/100 g.

Proximal Composition	WB (g/100 g)
Protein	17.31 ± 0.02
Fat	4.32 ± 0.41
Total dietary fibre (TDF)	36.81 ± 3.24
Carbohydrates (Including TDF)	61.93 ± 0.09
Ash	4.93 ± 0.10
Moisture	11.50 ± 0.20

**Table 2:** WB phenol characterization: Control, AUT100, AUT115 and AUT130. Phenol content is expressed as mg 100 g of WB.

Phenols	Control	AUT100	AUT115	AUT130
Caffeic acid O-hexoside	0.85 ± 0.02 a	1.11 ± 0.15 b	1.17 ± 0.12 b	1.06 ± 0.06 b
Apigenin-6/8-C-pentoside-8/6-C-hexoside i1	1.81 ± 0.05d	0.29 ± 0.09 a	0.37 ± 0.00 b	0.5 ± 0.04 c
Apigenin-6/8-C-pentoside-8/6-C-hexoside i2	2.25 ± 0.87d	0.18 ± 0.04 a	0.25 ± 0.02 b	1.36 ± 0.25 c
Apigenin-6-C-arabinoside-8-C-hexoside	0.52 ± 0.06 a	0.93 ± 0.05 c	0.76 ± 0.81 b	0.97 ± 0.20 c
Dihydroferulic acid	0.52 ± 0.06 a	0.76 ± 0.01 b	1.00 ± 0.48 b	0.83 ± 0.15 b
Ferulic acid	6.41 ± 0.08 a	7.21 ± 0.69 a	22.43 ± 0.46 b	24.13 ± 0.68 c

Values with different small letter show significant differences (*p* < 0.05) between autoclave treatments.

**Table 3:** Functional properties of autoclaved WB Control, AUT100, AUT115 and AUT130.

	WAC	OAC	WAI	WSI	SP	FC	FS	EA	ES	LGC
	(g/g)	(g/g)	(g/g)	(g/100g)	(g/g)	(mL/g)	(%)	(%)	(%)	(%)
Control	2.80 ± 0.06 b	2.62 ± 0.01 ab	4.22 ± 0.19 b	13.1 ± 0.9 b	3.9 ± 0.19 ab	5.1 ± 1.0 d	42 ± 7 a	49 ± 1 a	49 ± 3 a	15 ± 1.4 a
AUT100	2.80 ± 0.02 b	2.73 ± 0.04 c	4.21 ± 0.13 b	11.0 ± 0.2 a	4.0 ± 0.13 b	1.7 ± 0.3 b	72 ± 5 b	n.d.	n.d.	17 ± 1.4 a
AUT115	2.64 ± 0.08 a	2.66 ± 0.02 b	4.11 ± 0.03 ab	13.1 ± 0.3 b	3.8 ± 0.04 ab	3.6 ± 0.5 c	50 ± 11 a	47 ± 3 a	46 ± 3 a	16 ± 2.8 a
AUT130	3.05 ± 0.05 c	2.58 ± 0.01 a	3.95 ± 0.14 a	11.7 ± 0.4 a	3.7 ± 0.15 a	0.5 ± 0.0 a	100 ± 0 c	n.d.	n.d.	15 ± 1.4 a

WAC: water absorption capacity, OAC: oil absorption capacity, WAI: water absorption index, WSI: water solubility index, SP: swelling power, FC: foaming capacity, FS: foam stability, EA: emulsion activity, ES: emulsion stability and LGC: least gelation concentration. n.d: non detectable. Samples with different small letters show significant differences between autoclave treatments (*p* < 0.05).

**Table 4:** Viscosity and gelification of WB Control, AUT100, AUT115 and AUT130. G_1_′, G_1_′′ and tan (δ)_1_, represent elastic and viscous moduli and tangent at a frequency of 1 Hz. a, b and c are the equation exponents that are adjusted to model the law of potency from the experimental data (G′ (ω) = G1′ ωa; G′′ (ω) = G1′′ ωb; tan (δ) ω = tan (δ)_1_ ωc). τ_max_ represents the maximum tension in the viscoelastic lineal region (LVR) obtained from the tension screening. The cross-point is G1′ = G1′′.

	Control	AUT100	AUT115	AUT130
**Pasting properties**				
Pasting Temperature (°C)	70.3 ± 0.2 a	73.53 ± 0.2 b	73.6 ± 0.3 b	73.9 ± 0.0 b
Peak viscosity (Pa·s)	1.54 ± 0.03 c	1.44 ± 0.04 b	1.47 ± 0.01 bc	1.07 ± 0.00 a
Peak time (s)	676 ± 10 a	719 ± 60 b	784 ± 4 c	799 ± 17 c
Trough viscosity (Pa·s)	1.19 ± 0.03 b	1.30 ± 0.03 c	1.41 ± 0.01 d	1.07 ± 0.00 a
Breakdown (Pa·s)	0.35 ± 0.01 d	0.14 ± 0.01 c	0.06 ± 0.02 b	0.00 ± 0.00 a
Final Viscosity (Pa·s)	2.35 ± 0.05 ab	2.43 ± 0.03 b	2.62 ± 0.01 c	2.30 ± 0.01 a
Setback (Pa·s)	1.15 ± 0.03 a	1.13 ± 0.00 a	1.21 ± 0.00 b	1.24 ± 0.01 b
**Viscoelastic properties**				
G_1_′ (Pa)	867 ± 85 b	541 ± 107 a	775 ± 71 b	732 ± 3 ab
a	0.15 ± 0.01 a	0.15 ± 0.01 a	0.15 ± 0.00 a	0.15 ± 0.00 a
G_1_′′ (Pa)	179 ± 15 b	120 ± 22 a	169 ± 12 b	164 ± 0 b
b	0.28 ± 0.01 a	0.32 ± 0.00 b	0.29 ± 0.01 a	0.29 ± 0.00 a
tan (δ)_1_ (Pa)	0.206 ± 0.002 a	0.222 ± 0.003 b	0.219 ± 0.005 b	0.224 ± 0.000 b
c	0.130 ± 0.00 a	0.168 ± 0.01 b	0.137 ± 0.02 a	0.134 ± 0.00 a
τ _max_ (Pa)	3.7 ± 0.4 b	1.3 ± 0.1 a	1.2 ± 0.1 a	1.7 ± 0.4 a
Crosspoint (Pa)	41.4 ± 4.4 b	27.9 ± 0.0 a	31.5 ± 1.5 a	28.6 ± 0.3 a

Values with different small letters show significant differences between autoclave treatments (*p* < 0.05).

**Table 5:** Colorimetric parameters of WB Control, AUT100, AUT115, AUT130 and WB autoclaved at 130 °C with different particle size, >300, 300–106, and <106 µm.

	Control	>300 µm	300–106 µm	<106 µm	AUT100	AUT115	AUT130
L*	63.48 ± 0.71 c	64.83 ± 0.70 d	74.69 ± 0.52 e	83.55 ± 0.39 f	61.36 ± 1.5 b	60.46 ± 1.1 a	60.14 ± 0.99 a
a*	7.50 ± 0.33 d	6.92 ± 0.34 c	4.41 ± 0.14 b	2.60 ± 0.06 a	6.97 ± 0.41 c	6.85 ± 0.5 c	7.40 ± 0.20 d
b*	17.94 ± 0.62 d	17.26 ± 0.52 c	16.10 ± 0.19 b	13.03 ± 0.18 a	18.58 ± 0.79 e	18.83± 0.49 ef	19.26 ± 0.37 f
Δc	0.85 ± 044 bc	0.85 ± 0.40b c	0.53 ± 0.39 ab	0.34a ± 0.24 a	1.54 ± 0.66 d	1.06 ± 0.61 c	0.96 ± 0.38 c
Hue	1.17 ± 0.01 a	1.19 ± 0.01 b	1.30 ± 0.01 e	1.37 ± 0.01 f	1.21 ± 0.02 cd	1.22 ± 0.02 d	1.2 ± 0.01 c
Croma	19.45 ± 0.67 d	18.59 ± 0.60 c	16.70 ± 0.20 b	13.28 ± 0.18 a	19.85 ± 0.80 de	20.50 ± 0.42 e	20.64 ± 0.34 f

Values with different small letters show significant differences between treatments (*p* < 0.05).

## Supplementary Materials

The following are available online at https://www.mdpi.com/2304-8158/9/6/781/s1, Table S1. Chromatographic and mass spectrum data of the phenolic compounds identified in WB. [M-H]^−^ (m/z):, MS (m/z):, R.T: Retention time, Figure S1: Pasting profiles of autoclaved wheat bran. Control, AUT100, AUT115 and AUT130. The temperature profile is represented in the second axis, Figure S2: Viscoelastic moduli (elastic, G′, and viscous, G′′) evolution vs frecuency of autoclaved wheat bran. Filled lines represent the elastic modulus, lines with gaps represent the viscous modulus. Control, AUT100, AUT115 and AUT130.
