# Supplementary material for: Application of Autoclave Treatment for Development of a Natural Wheat Bran Antioxidant Ingredient
Source: Foods. 2020 Jun 12;9(6):781. doi: 10.3390/foods9060781 (PMC7353647; doi:10.3390/foods9060781)
Supplement: Supplementary file 1 [file foods-09-00781-s001.zip › foods-818069-supplementary.pptx]

## Slide 1
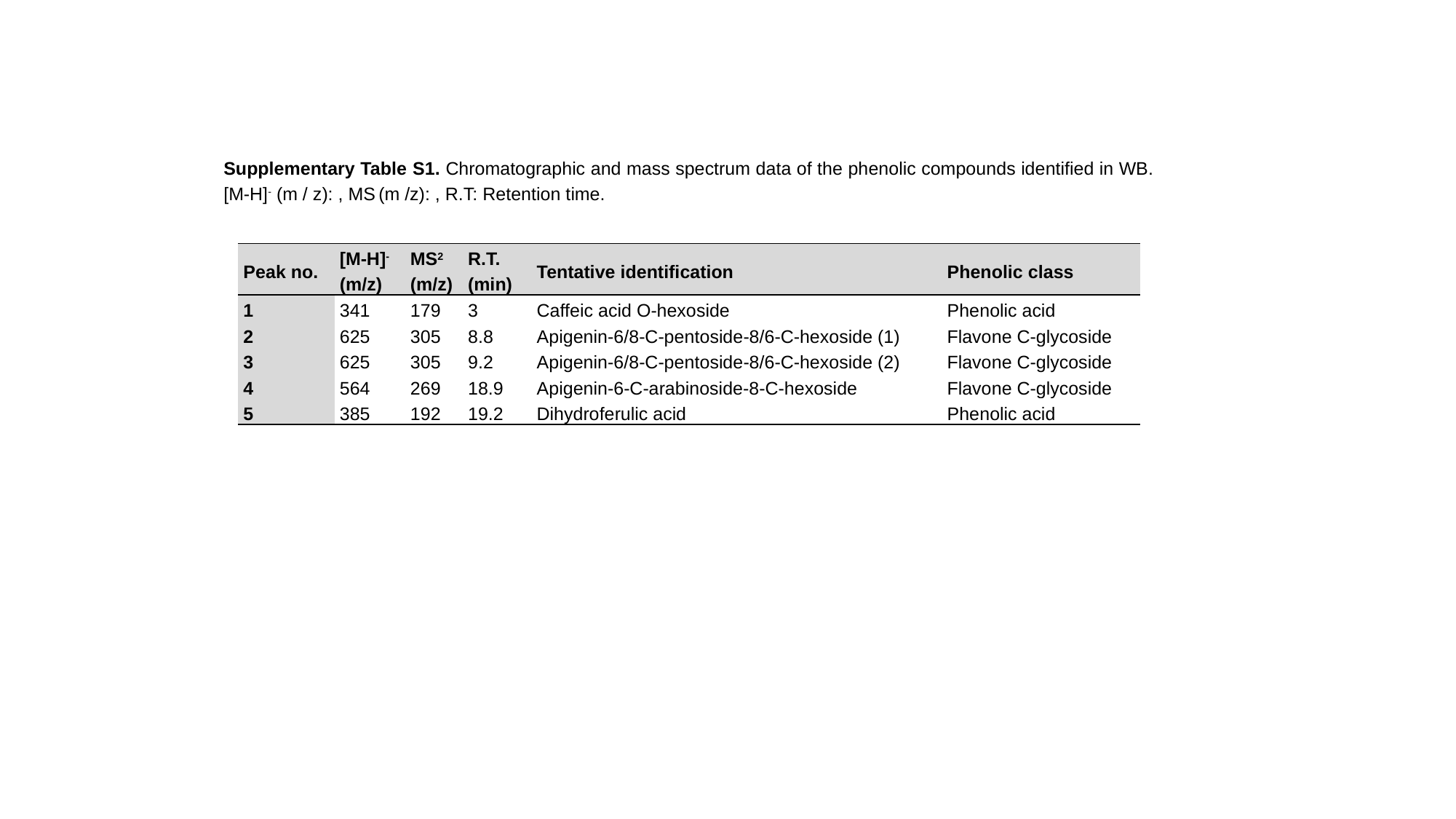

Supplementary Table S1. Chromatographic and mass spectrum data of the phenolic compounds identified in WB. [M-H]- (m / z): , MS (m /z): , R.T: Retention time.
| Peak no. | [M-H]- (m/z) | MS2 (m/z) | R.T. (min) | Tentative identification | Phenolic class |
| --- | --- | --- | --- | --- | --- |
| 1 | 341 | 179 | 3 | Caffeic acid O-hexoside | Phenolic acid |
| 2 | 625 | 305 | 8.8 | Apigenin-6/8-C-pentoside-8/6-C-hexoside (1) | Flavone C-glycoside |
| 3 | 625 | 305 | 9.2 | Apigenin-6/8-C-pentoside-8/6-C-hexoside (2) | Flavone C-glycoside |
| 4 | 564 | 269 | 18.9 | Apigenin-6-C-arabinoside-8-C-hexoside | Flavone C-glycoside |
| 5 | 385 | 192 | 19.2 | Dihydroferulic acid | Phenolic acid |

## Slide 2
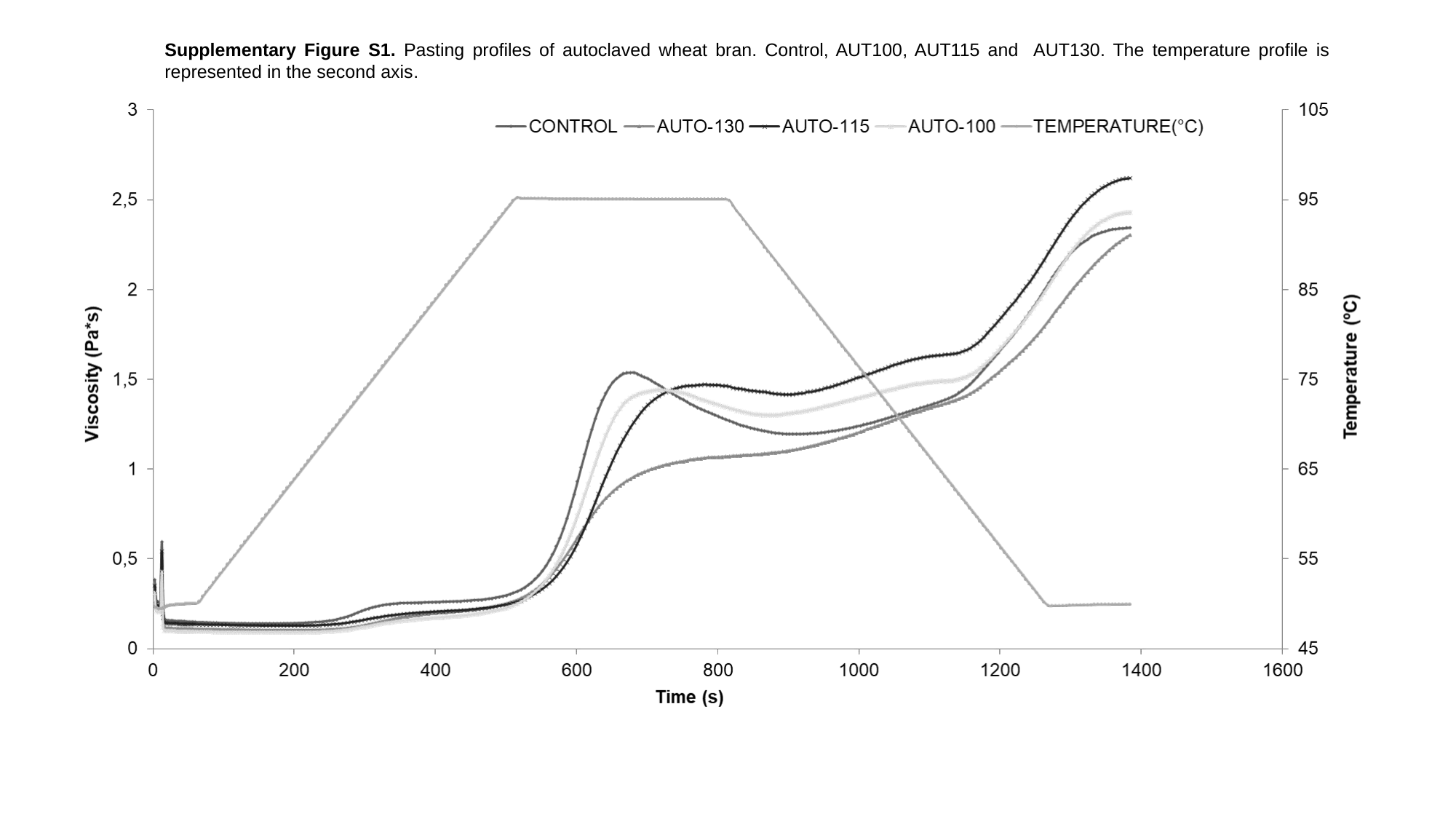

Supplementary Figure S1. Pasting profiles of autoclaved wheat bran. Control, AUT100, AUT115 and AUT130. The temperature profile is represented in the second axis.

## Slide 3
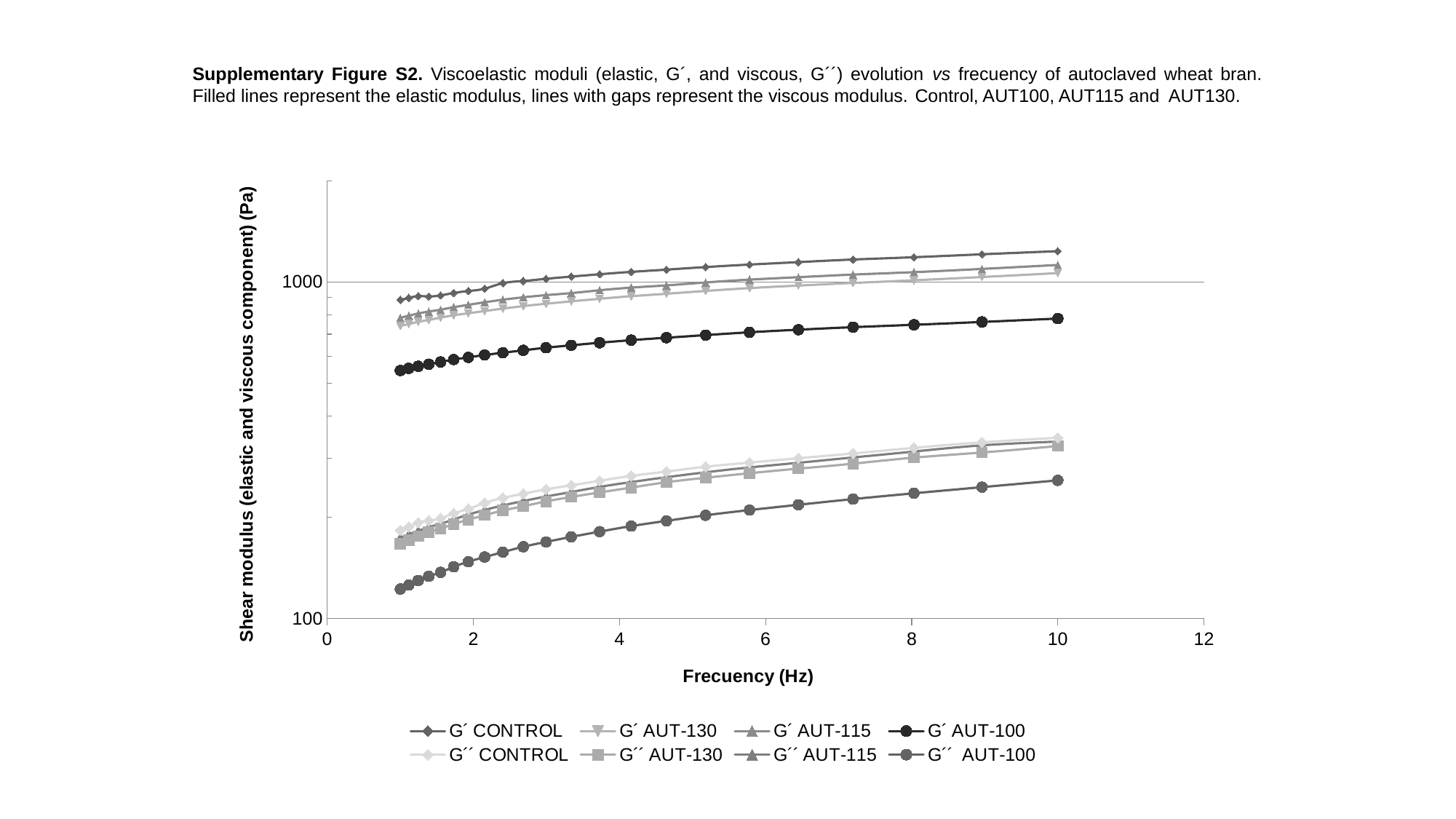

Supplementary Figure S2. Viscoelastic moduli (elastic, G´, and viscous, G´´) evolution vs frecuency of autoclaved wheat bran. Filled lines represent the elastic modulus, lines with gaps represent the viscous modulus. Control, AUT100, AUT115 and AUT130.
### Chart
| Category | G´ CONTROL | G´ AUT-130 | G´ AUT-115 | G´ AUT-100 | G´´ CONTROL | G´´ AUT-130 | G´´ AUT-115 | G´´ AUT-100 |
|---|---|---|---|---|---|---|---|---|
